# Colorectal endometriosis: ample data without definitive recommendations

**DOI:** 10.52054/FVVO.13.1.006

**Published:** 2021-03-31

**Authors:** GN Moawad, JS Klebanoff, N Habib, S Bendifallah

**Affiliations:** Department of Obstetrics and Gynecology, The George Washington University, Washington, DC; The Center for Endometriosis and Advanced Pelvic Surgery, Washington, DC; Department of Obstetrics and Gynecology, Main Line Health, Wynewood, PA; Department of Obstetrics and Gynecology, Francois Quesnay Hospital, Mantes-la-Jolie, France; Department of Gynaecology and Obstetrics, Tenon University Hospital, Assistance Publique des Hopitaux de Paris (AP-HP), Sorbonne University, France

**Keywords:** Colorectal endometriosis, shaving, disc excision, segmental resection

## Abstract

The preoperative work-up and optimal surgical approach to colorectal endometriosis is a highly studied topic lacking definitive recommendations. Synthesis of the available data can be extremely challenging for surgeons due to the heterogeneity of existing comparisons, a variety of studied surgical outcomes, and a predominant focus on operative complications. While these considerations are extremely important for surgeons performing such complex gynaecologic surgery there is still much to be desired with regards to evidence based guidelines for the preoperative assessment and surgical technique for colorectal endometriosis. Having an established guideline stating in which clinical situations endometriosis surgeons should performing rectovaginal shaving, versus discoid excision, versus segmental resection would be extremely important for all pelvic surgeons, even those operating in high-volume centres dedicated to the surgical management of complex endometriosis. This perspective highlights the shortcomings of the available data and attempts to create an algorithm surgeons can follow when performing surgery for colorectal endometriosis. This algorithm is based on our expert opinion after synthesising available data.

## Perspective

There has long been debate regarding the most appropriate preoperative imaging study and surgical approach for management of deeply infiltrating endometriosis (DIE) of the bowel. As if this were not enough, we also lack a consensus definition on how to classify DIE. A commonly accepted definition for DIE is when the disease penetrates the peritoneal surface by more than 5 mm (Darwish and Roman, 2016). Unfortunately, regarding complex pelvic surgery no clear guidelines exist to provide a systematic and reproducible approach to the preoperative investigation and planning of surgery for DIE (Habib et al., 2020). This lack of definitive instruction makes it challenging for endometriosis surgeons to consistently practice evidence-based medicine. Knowing exactly when a transvaginal ultrasound (US) will suffice before surgery as compared to more sophisticated (and expensive) tests such as a magnetic resonance image (MRI) or barium enema is difficult to decipher (Indrielle-Kelly et al., 2020). Deciding on which preoperative imaging technique to utilise requires considering the entire patient, the location and extent of disease, and previous surgical history. We lack high quality data comparing commonly used preoperative imaging studies to help plan for surgery. US has been shown to be sensitive and specific in the hands of expert sonographers, but unfortunately it’s sensitivity and specificity is user dependent (Ros et al., 2017). MRI has superior sensitivity and specificity for detecting DIE, especially in areas outside of the pelvis, and can help coordinate a surgical approach (Thomassin- Naggara et al., 2020). With the ability of MRI to accurately identify DIE of the rectosigmoid, appropriate preoperative counseling and planning may help to reduce patient morbidity. Correct identification of deep colorectal disease should prompt a multidisciplinary approach to care with colorectal surgery as well as preoperative bowel preparation for surgery. The multidisciplinary approach to colorectal endometriosis often involves gynaecologic surgeons, colorectal surgeons, and radiologists.

When it comes to the specific surgical technique conflicting data between the three most widely studied surgical techniques; shaving, disc excision, and segmental resection, make it challenging for pelvic surgeons to know which technique is most suitable for an individual patient (Bendifallah et al., 2020; Roman et al., 2018). Most studies comparing these surgical approaches focus on the multitude of possible postoperative complications and patient outcomes (Kondo et al., 2011; Oliveira et al., 2016). It can also be very difficult to analyse and compare data from different surgical specialties and between surgeons with varying levels of expertise. Despite our best efforts through rigorous study and ample data endometriosis surgeons are still left without definitive answers.

Certain themes have become clinically apparent, primarily that when approaching complex endometriosis patients with DIE of the bowel a multidisciplinary approach is paramount to an individual’s approach (Nezhat et al., 2018; Graham et al., 2019). Ultimately, this multidisciplinary team often includes an experts review, or performance, of preoperative imaging, a pelvic surgeon trained in advanced surgery for endometriosis, and a colorectal surgeon. It is also recommended that for gynaecologic surgeons not comfortable or trained in the practice of bowel surgery that a colorectal surgeon knowledgeable about the disease be present for these portions of the surgery. However, for the more pressing questions of when to perform a disc excision versus a segmental resection, or when disease can be treated with shaving alone we are left relying on our own clinical judgment.

Understanding the commonly used surgical terminology may be challenging for pelvic surgeons without specific training in rectovaginal DIE. Specifically, differentiating between rectal shaving and a disc excision can be difficult for those unfamiliar with these specific techniques. The commonly used term ‘shaving’ has been previously defined (Reich et al., 1991). In the most basic sense this technique involves separating the endometriosis nodule from the anterior rectum to delineate the rectovaginal space (Donnez and Roman, 2017). The DIE nodule is then excised even if the lumen of the bowel is inadvertently entered, however, this requires repair. A disc excision is often a multistep process with or without the assistance of a transanal end-to-end anastomosis stapler (EEA stapler^TM^). The first step involved in successful disc excision involves rectal shaving. However, when residual disease remains on the bowel, which is assessed in real time through instrument probing, appearance, and clinical judgement, then excision of the diseased area must be completed. This is done by either full thickness excision laparoscopically with suture repair, or the diseased area is excised using EEA staplersTM with laparoscopic assistance (Donnez and Roman, 2017). The purpose of the laparoscopic assistance is to bring the endometriosis nodule down into the path of the EEA stapler^TM^, this is often done by placing a suture through the nodule and guiding the nodule into the opening of the stapler once deployed.

There are many potential complications associated with surgery for DIE of the bowel. For surgeons performing segmental resections special attention to sparing the pelvic nerves will minimise impaired functionality following surgery. Common complications that have also been highly scrutinised include rates of anastomotic leakage (AL) and rectovaginal fistula (RVF), as well as voiding dysfunction following surgery for bowel endometriosis (Vesale et al., 2020). A recent large systematic review and meta-analysis found that shaving was associated with a lower incidence of both AL and RVF compared to disc excision and segmental resection, and this reached statistical significance (Bendifalla et al., 2021). That review found no statistically significant difference in these complications between disc excision and segmental resection (Bendifalla et al., 2021). Similar results were found by Vigueras Smith et al. (2020) in their review of the literature assessing these complications following shaving, disc excision, and segmental resection. It is important to note operative time as Vigueras el al found that increased operative time is associated with increased patient morbidity. While segmental resection is associated with the longest total operative time and bowel shaving is associated with the lowest total operative time, no studies have been powered to detect a significant difference between technique with regard to operative time and associated complications. It is our opinion that surgeons should consider the surgical approach they are most efficient in performing when considering their surgical approach to colorectal endometriosis. Often patients with DIE are undergoing surgery to improve fertility rates (Breteau et al., 2020).

What must also be considered is that postoperative complications can either delay patients fertility plans, or significantly reduce the likelihood of a clinical pregnancy in the future. Thus, in fertility patients undergoing surgery for DIE considerations must be made to minimise complication rates following surgery as postoperative complications can have a significant impact even following complete resolution of the complication. Surgeons must also consider how the effects of pelvic surgery can impact future pregnancy, specifically with regards to adhesion formation, tubal patency, and ovarian reserve.

We must, however, acknowledge the inherent bias that exists within all of the reviews done comparing surgical technique because no study includes women that have the same size endometriosis nodule. Thus, it is reasonable to conclude that excision of larger nodules, often by disc excision or segmental resection, is associated with more innate risks than shaving of smaller nodules. Despite the rigorous study that has been dedicated to this topic it remains challenging to draw firm conclusions from the available data. A major limitation present is the heterogeneity of studies included in these reviews (Balla et al., 2018; Bendifalla et al., 2020; Donnez and Roman, 2017). Understandably, it is very difficult to account for all patient and surgical variables including stage of disease, location and size of endometriosis nodules, patient comorbidities, surgeon experience and surgical team, indication for surgery especially in case of infertility, and technique for excision to name only a few. Definitions remain inconsistent throughout different studies as AL and RVF are not always clearly defined. Data from both endometriosis patients and colorectal cancer patients are often analysed together in large reviews, which again introduces bias and heterogeneity into the findings (Vigueras Smith et al., 2020). What we as readers are left with following these large scale narrative and systematic reviews really amounts to expert opinion on a complex topic. However, the opinion focuses mostly on differences in rates of complications following surgery and less often we are left with expert instruction, which is what is truly desired. For endometriosis surgeons operating at lesser volumes than those working out of centres dedicated to deeply infiltrating disease of the rectosigmoid or for those surgeons without a dedicated colorectal team an algorithm is needed providing instruction on when to consider a particular surgical technique. One area of investigation that may help surgeons provide evidence-based care that receives less attention than surgical complications is patient outcomes. Investigating whether patient satisfaction and improvement in quality of life differs between surgical techniques one could argue is equally, if not more important, than complication rates. Evidence has suggested that segmental resection is associated with poorer functional outcomes than either shaving or disc excision, however, much more robust data is needed regarding the impact on health related quality of life between these surgical techniques (Donnez and Roman, 2017). It is critical to know if one of these surgical techniques is associated with a significantly longer pain free interval, improved emotional well-being, sense of empowerment, and self-esteem than another.

Another major issue is the choice of infertility treatment for women with DIE, and especially for those with colorectal endometriosis. Similarly, due to the absence of a high level of evidence, and despite international guidelines, there is currently no consensus about the best strategies.

The European Society of Human Reproduction and Embryology (ESHRE) guidelines state that the effectiveness of surgical excision of deep nodular lesions before assisted reproductive technology (ART) in women with endometriosis-associated infertility is not well established in terms of reproductive outcome. In a literature review, Cohen et al. (2020) reported a pregnancy rate (PR) of 37.9% after ART in infertile women with in situ bowel endometriosis and of 35% after ART in infertile women after surgical removal of colorectal endometriosis. In this setting, most recently, the impact comparison of first-line assisted reproductive technology (ART; intracytoplasmic sperm injection [ICSI]-IVF) and first-line colorectal surgery followed by ART on fertility outcomes in women with colorectal endometriosis-associated infertility has been reported. The authors suggested that that first-line surgery is correlated with higher pregnancy rates, live birth rates (LBR), and cumulative LBRs than first-line ART in the whole population, for women with good prognosis and with an anti- mullerian hormone (AMH) level < 2 ng/mL. However, these results must be interpreted with caution and debated according to complications. In practice the real challenge is to identify women who will benefit from first-intention ART and those who will benefit from first-line surgery especially due to the risk of complication.

In analysing the most recent, and to date the largest, reviews of surgery for DIE of the bowel (Bendifalla et al., 2021; Vigueras Smith et al., 2020) we have synthesised the following expert recommendations. We must reiterate that the recommendations that follow are based on our opinion of the available data. Bowel shaving is associated with an overall lower rate of complication than either disc excision or segmental resection. In general bowel shaving should be attempted for single endometriosis nodules less than 3 cm in size regardless of location. For lesions larger than 3 cm shaving is still a reasonable option, however, it is also appropriate to perform a disc excision utilising either a trans-anal stapler or a hand-sewn repair. Evidence also suggests that for nodules larger than 5 cm a double disc excision is a safe and feasible option for surgeons familiar with this technique (Namazov et al., 2020). When multiple nodules larger than 3 cm are infiltrating the bowel a segmental resection is preferred over multiple different disc excisions, however, this recommendation should not supersede clinical judgment. For endometriosis nodules within 8 cm of the anal verge we recommend either a shaving technique, if feasible based on size, or disc excision as segmental resection has a higher risk of postoperative complications (low anterior resection syndrome in particular) when this close to the anal verge.

Pelvic surgery for DIE of the bowel is a complex undertaking with many patient, surgical, and health system level variables to consider. We would like to reiterate that good scientific evidence suggests that patient outcomes are optimised when pelvic surgery for DIE of the bowel is performed in a multidisciplinary fashion in high volume centers focused on this disorder (Graham et al., 2019; Nezhat et al., 2018). While rigorous study has been dedicated to the complex topic of the preferred surgical modality for DIE of the bowel there are serious limitations of the available data. We lack guidelines to help surgeons provide evidence-based care for the patients they serve. We have attempted here in this perspective to synthesise the available data and provide some simplified guidance for surgeons in need of a blueprint when approaching DIE of the bowel. These recommendations should in no way replace clinical judgment; however, we hope may help clinicians decide on which specific surgical technique to utilise in which individual clinical situation based on the available evidence.

## References

[1] Balla A, Quaresima S, Subiela JD (2018). Outcomes after rectosigmoid resection for endometriosis: a systematic literature review.. Int J Colorectal Dis.

[2] Bendifallah S, Puchar A, Vesale E (2021). Surgical outcomes after colorectal endometriosis: a systematic review and meta-analysis.. J Minim Invasive Gynecol.

[3] Breteau P, Chanavaz-Lacheray I, Rubod C (2020). Pregnancy rates after surgical treatment of deep infiltrating endometriosis in infertile patients with at least 2 previous in vitro fertilization or intracytoplasmic sperm injection failures.. J Minim Invasive Gynecol.

[4] Cohen J, Thomin A, Mathieu D’Argent E (2014). Fertility before and after surgery for deep infiltrating endometriosis with and without bowel involvement: a literature review.. Minerva Gynecol.

[5] Donnez O, Roman H (2017). Fertil Steril. Choosing the right surgical technique for deep endometriosis: shaving, disc excision, or bowel resection.

[6] Darwish B, Roman H (2016). Surgical treatment of deep infiltrating rectal endometriosis: in favor of less aggressive surgery.. Am J Obstet Gynecol.

[7] Graham A, Chen S, Skancke M (2019). A review of deep infiltrative colorectal endometriosis treated robotically at a single institution. Int J Med Robot Comput Assist Surg.

[8] Habib N, Centini G, Lazzeri L (2020). Bowel endometriosis: current perspectives on diagnosis and treatment.. Int J Womens Health.

[9] Indrielle-Kelly T, Frühauf F, Fanta M (2020). Diagnostic accuracy of ultrasound and MRI in the mapping of deep pelvic endometriosis using the international deep endometriosis analysis (IDEA) consensus.. Biomed Res Int.

[10] Kondo W, Bourdel N, Tamburro S (2011). Complications after surgery for deeply infiltrating pelvic endometriosis.. BJOG.

[11] Moawad GN, Tyan P, Abi Khalil ED (2018). Multidisciplinary resection of deeply infiltrative endometriosis.. J Minim Invasive Gynecol.

[12] Moawad GN, Wu C, Klebanoff JS (2021). Pelvic neuroanatomy: an overview of commonly encountered pelvic nerves in gynecologic surgery.. J Minim Invasive Gynecol.

[13] Namazov A, Kathurusinghe S, Marabha J (2020). Double disk excision of large deep endometriosis nodules infiltrating the low and mid rectum: a pilot study of 20 cases.. J Minim Invasive Gynecol.

[14] Nezhat C, Li A, Falik R, Copeland D (2018). Bowel endometriosis: diagnosis and management.. Am J Obstet Gynecol.

[15] Oliveira MAP, Pereira TRD, Gilbert A (2016). Bowel complications in endometriosis surgery.. Best Pract Res Clin Obstet Gynaecol.

[16] Reich H, McGlynn F, Salvat J (1991). Laparoscopic treatment of cul-de-sac obliteration secondary to rectocervical deep fibrotic endometriosis.. J Reprod Med.

[17] Roman H, Bubenheim M, Huet E (2018). Conservative surgery versus colorectal resection in deep endometriosis infiltrating the rectum: a randomized trial.. Hum Reprod.

[18] Ros C, Martinez-Serrano M, Rius M (2017). Bowel preparation improves the accuracy of the transvaginal ultrasound in the diagnosis of rectosigmoid deep infiltrating endometriosis: a prospective study.. J Minim Invasive Gynecol.

[19] Thomassin-Naggara I, Lamrabet S, Crestani A (2020). Magnetic resonance imagining classification of deep pelvic endometriosis: description and impact on surgical management.. Hum Reprod.

[20] Vigueras Smith A, Sumak R, Cabrera R (2020). Bowel anastomosis leakage following endometriosis surgery: an evidence based analysis of risk factors and prevention techniques.. Facts Views Vis Obgyn.

[21] Vesale E, Roman H, Moawad G (2020). Voiding dysfunction after colorectal surgery for endometriosis: a systematic review and meta-analysis.. J Minim Invasive Gynecol.

